# Optimisation of an oviposition protocol employing human chorionic and pregnant mare serum gonadotropins in the Barred Frog *Mixophyes fasciolatus* (Myobatrachidae)

**DOI:** 10.1186/1477-7827-10-60

**Published:** 2012-08-21

**Authors:** John Clulow, Simon Clulow, Jitong Guo, Andrew J French, Michael J Mahony, Michael Archer

**Affiliations:** 1 School of Environmental and Life Sciences, University of Newcastle, Callaghan Drive, Callaghan, NSW, 2308, Australia; 2 Inner Mongolia Saikexing Reproductive Biotechnology Co., Ltd. 6 F, Mengniu Dairy R&D Center, Shengle Economic Zone of Helingeer County, Hohhot, 011517, Inner Mongolia, People's Republic of China; 3 Centre for Animal Biotechnology, Faculty of Veterinary Science, The University of Melbourne, Parkville, 3010, VIC, Australia; 4 School of Biological, Earth and Environmental Sciences, University of New South Wales, Sydney, 2052, Australia

**Keywords:** Amphibian, Mixophyes, Ovulation, Oviposition, Assisted reproduction, hCG, PMSG

## Abstract

**Background:**

Protocols for the hormonal induction of ovulation and oviposition are essential tools for managing threatened amphibians with assisted reproduction, but responses vary greatly between species and even broad taxon groups. Consequently, it is necessary to assess effectiveness of such protocols in representative species when new taxa become targets for induction. The threatened genus *Mixophyes* (family Myobatrachidae) has amongst the highest proportion of endangered species of all the Australian amphibians. This study developed and optimised the induction of oviposition in a non-threatened member of this taxon, the great barred frog (*Mixophyes fasciolatus*).

**Methods:**

Gravid female *M. fasciolatus* were induced to oviposit on one or more occasions by administration of human chorionic gonadotropin (hCG) with or without priming with pregnant mare serum gonadotropin (PMSG). Treatments involved variations in hormone doses and combinations (administered via injection into the dorsal lymph sacs), and timing of administration. Pituitary homogenates from an unrelated bufonid species (*Rhinella marina*) were also examined with hCG.

**Results:**

When injected alone, hCG (900 to 1400 IU) induced oviposition. However, priming with two time dependent doses of PMSG (50 IU, 25 IU) increased responses, with lower doses of hCG (200 IU). Priming increased response rates in females from around 30% (hCG alone) to more than 50% (p = 0.035), and up to 67%. Increasing the interval between the first PMSG dose and first hCG dose from 3 to 6 days also produced significant improvement (p<0.001). Heterologous pituitary extracts administered with hCG were no more effective than hCG alone (p = 0.628).

**Conclusions:**

This study found that *M. fasciolatus* is amongst the few amphibian species (including *Xenopus* (*Silurana*) and some bufonids) that respond well to the induction of ovulation utilising mammalian gonadotropins (hCG). The optimal protocol for *M. fasciolatus* involved two priming doses of PMSG (50 IU and 25 IU) administered at 6 and 4 days respectively, prior to two doses of hCG (100 IU), 24 hours apart. This study is also the first to demonstrate in an amphibian species that responds to mammalian gonadotropins that an increase in the ovulation rate occurs after priming with a gonadotropin (PMSG) with FSH activity.

## Background

Pituitary extracts have been widely used for many decades as an effective and practical tool for obtaining viable oocytes from gravid amphibians [1-6] since the first bioassays demonstrated the effects of pituitary extracts on amphibian ovulation. Pituitary extracts from the same (homologous) species are the most effective at inducing ovulation [2]. Homologous pituitaries are presumably the most suitable and potent source of LH activity [2] for the induction of ovulation because of sequence variation in the active β unit of the gonadotropins [7] that impacts on hormone-receptor specificity, an effect that is likely to increase with phylogenetic distance. Heterologous pituitary extracts from different amphibian species are a “second best” source of LH activity [5] but still (with exceptions such as *Xenopus*, Table 1) much more effective than phylogenetically distant mammalian gonadotropins [2,8]. However, disadvantages of using heterologous or homologous pituitary extracts as a routine tool for inducing ovulation and oviposition in gravid females include individual, gender and seasonal variability in FSH and LH activity [6], availability of a source of pituitaries, and the cost and inconvenience of the collection process. In the case of endangered species, the potential for transfer of pathogens in raw extracts [9] as well as the inappropriateness of using rare or endangered animals to obtain pituitary extracts argues for alternative induction protocols. 

**Table 1:** **Published protocols for the induction of oviposition in*Xenopus***

**Species**	PMSG priming dose (FSH activity)*	hCG priming dose (LH activity)*	“Ovulatory” hCG (dose or source) on Day 1**	Oviposition***	**References**
*Xenopus laevis*			Human pregnant urine	Time not stated	[2,10]
*X. laevis*			90 IU	Time not stated	[11]
*X. laevis*			100-200 IU (< 100 g bwt)	+ 6–12 hrs	[12]
			200–600 IU (> 100 g bwt)		
*X. laevis*			300 IU	Time not stated	[13]
*X. laevis*			500 IU	+ 8–10 hrs	[14]
*X. laevis*			500 IU	Time not stated	[15]
*X. laevis*			500-700 IU	Day 1	[16]
*X. laevis*			750 IU	+ 8–12 hrs	[17]
*X. laevis*			900 IU	+ 8–10 hrs	[18]
*X. laevis*			1000 IU	+ 10 hrs	[19]
*X. laevis*			1000 IU	+ 18 hrs	[20]
*X. laevis*		50 IU, Day −14 to −5 (early ovulation if priming after day −5; priming effective up to 1 month)	500-800 IU	+ 9–14 hrs	[21]
*X. laevis*		50-200 IU, Day 1 (−8 hrs)	300-500 IU; Day 1 (0 hrs)	+ 8 hrs	[22]
*X. laevis*	40 IU, Day −4 to −3		250-500 IU	+ 6–8 hrs	[23]
*X. laevis*	50 IU, Day?		700 IU	Time not stated	[24]
*X. laevis*	50 IU, Day −5 to −3		500 IU	+ 12–14 hrs	[25]
*X. laevis*	50 IU, Day −17 25 IU, Day −14		500 IU (hCG inducible over 14 days from second PMSG)	+ 16–18 hrs	[26]
*X. laevis*	100 IU, Day −1		500 IU	Day 2, avg 68%	[27]
*X. tropicalis*		20 IU, Day −2	100 IU	Natural pairing	[28]
*X. tropicalis*	15 IU, Day −4 to −3		150 IU	+ 4 hrs	[29]

Protocols for the induction of oviposition of *Xenopus* (*X. laevis* and *X. (Silurana) tropicalis*) utilising hCG with, or without, priming with mammalian gonadotropins with FSH or LH activity. * Day of administration of priming dose of PMSG or hCG expressed as days before the main “ovulatory” hCG dose (expressed as negative values). ** Day of administration of “ovulatory” hCG dose expressed as Day 1 to facilitate comparison between studies listed in Tables 1 and 2; hCG administered as either reconstituted or synthetic hCG (dose in IU) or as direct injection of pregnant human urine (dose unknown) *** Time or day of oviposition (generally, hours after the main “ovulatory” dose of hCG on Day 1). Species names are from original papers, however, *Xenopus tropicalis* is now known as *Silurana tropicalis*.

One great advantage of working with *Xenopus spp* (*X. laevis* and *X. (Silurana*) tropicalis) in reproductive and developmental biology studies involving amphibians has been the ease with which ovulation of fertile eggs can be induced with mammalian gonadotropic hormones, especially hCG as a source of LH activity [8] (Table 1). *Xenopus* gained widespread attention in the 1930s [10] providing the first effective, large scale pregnancy test for women because of the reliability of its ovulatory response to hCG administration (an application that persisted for many years). This turned out to be an aspect of Xenopus biology shared with few other amphibians (Table 2). The poor response to mammalian gonadotropins in most species (Table 2) and the desire to move away from pituitary extracts for reasons outlined above has led to the development of protocols based on other parts of the hypothalamic-pituitary-gonadal axis. These include gonadotropic releasing hormones (GnRH) to stimulate release of endogenous gonadotropins [30-32], progesterone to directly induce oocyte maturation and germinal vesicle breakdown [33,34], and dopamine antagonists [35,36], as well as various combinations of these with or without gonadotropins (for recent reviews and discussions see [9,36,37]). Nevertheless, as a first step to inducing ovulation in a novel target species, it is reasonable to test the use of synthetic mammalian gonadotropins, to determine whether these are an effective and inexpensive means of obtaining viable eggs, as is the case in *Xenopus*. 

**Table 2:** **Results of attempts to induce oviposition in amphibians other than*Xenopus*with hCG**

**Species**	**Priming or co-administered agent**	hCG [dose or source]*	Oviposition**	**References**
*Bufo americanus*		Human pregnant urine	None	[2]
*B. americanus*	No priming	100 IU, Day 1	None	[38]
	No priming	400-1000 IU, Day 1	about 60%, Day 2	
	Priming Day −2 [LH 50 ug, or LHRH 50 ug, or eCG 50 IU, or hCG 50 IU]	500 IU, Day 1	≤ 60%, Day 2	
*B. arenarum*		Human pregnant urine	None	[2]
*B. baxteri*	4.0 ug LHRHa, Day 1 [no priming]	500 IU, Day 1	None	[35]
	0.8 ug LHRHa, Day −4	100 IU, Day −4	70%, Day 1-2	
	4.0 ug LHRHa, Day 1	500 IU, Day 1		
		[total = 600 IU]		
	4.0 ug LHRHa, Day −6	500 IU, Day −6	80%, Day 1-2	
	0.8 ug LHRHa, Day −4	100 IU, Day −4		
	4.0 ug LHRHa, Day 1	500 IU, Day 1		
		[total = 1100 IU]		
*B. calamita*		Human pregnant urine	None	[2]
*B. fowleri*		Human pregnant urine	+	[2]
*B. fowleri*	60 ug LHRHa, 5 mg progesterone, 0.25 mg pimozide, Day 1	500 IU, Day 1	85%, Day 1-2	[39]
	4 ug LHRHa, Day −1	500 IU, Day −1	29%, Day 1-2	
	4 ug LHRHa, Day 1	500 IU, Day 1		
		[total = 1000 IU]		
*B. vulgaris*		Human pregnant urine	None	[2]
*Eleutherodactylus coqui*		25-140 IU, Day 1	None	[40]
		165-200 IU, Day 1	Day 2, weak response (2/6 females)	
*Hyla aurea*	Pregnant mare serum	-	+	[2]
*Litoria aurea*	5 ug LHRHa, Day −4.5	300 IU, Day 1	Weak; 1% of normal egg release (2/5 females)	[41]
	5 ug LHRHa, Day −1.5			
	10 ug LHRHa, Day −0.5			
	20 ug LHRHa, Day 1			
*Litoria moorei*	No benefit of including progesterone	100-200 IU, Day −2	Weak; few eggs in 1/14 females	[41]
		500–750 IU, Day 1		
*Litoria raniformis*	10 ug LHRHa, Day −4	500 IU, Day −4	None	[42]
	10 ug LHRHa, Day −2	500 IU, Day −2		
	10 ug LHRHa, Day 1	500 IU, Day 1		
*Rana catesbeiana*		Human pregnant urine	+	[2]
*R. catesbeiana*	Pregnant mare serum	-	+	[2]
*R. clamitans*		Human pregnant urine	None	[2]
*R. temporaria*		Human pregnant urine	None	[2]
*R. esculenta*		Human pregnant urine	None	[2]
*R. pipiens*		Human pregnant urine	None	[2]
*R. pipiens*	Pregnant mare serum	-	None	[2]
*R. sevosa*	3-4 ug LHRH, Day −3	100 IU, Day −3	+	[37]
	15–20 ug LHRH, Day 1	500 IU, Day 1		
*R. vulgaris*		Human pregnant urine	None	[2]
**Urodeles**				
*Ambystoma tigrinum*		Human pregnant urine	+	[2]
*Triturus pyrrogaster*		Human pregnant urine	+	[2]
*T. torosus;*	Pregnant mare serum	-	None	[2]
*T. similans;*				
*T. rivularis*				
*T. viridescens*		Human pregnant urine	+	[2]

Results of attempts to induce oviposition utilising hCG administration in amphibian species other than *Xenopus* with, or without, priming with hCG or other agents. * Day of final hCG injection expressed as Day 1, with administration of compounds on days prior to Day 1 expressed as negative values (to facilitate comparison between studies listed in Tables 1 and 2); hCG administered as either reconstituted or synthetic hCG (dose in IU) or as direct injection of pregnant human urine (dose unknown). ** Oviposition: “+” = oviposition recorded; “None” = no oviposition recorded. Data from [2] for PMSG only inductions (no hCG) for some *Rana* species also shown. Species names are from original papers, however, some have since been renamed e.g. *Rana pipiens* now *Lithobates pipiens* by some [43].

Global declines since the 1960s have resulted in amphibians experiencing the highest rate of decline and extinction of any vertebrate class over that period [44-46]. This loss of amphibian biodiversity is primarily a function of a global pandemic of chytridiomycosis. Amongst Australian frogs, the genus *Mixophyes* (commonly known as the Barred Frogs) is amongst the most threatened genera. Currently, >40% (3 out of 7) of extant *Mixophyes* species are listed as vulnerable or endangered under Australian federal and state legislation, as well as the IUCN Red List. In the state of New South Wales, this figure is as high as 75% (3 out of 4) species of *Mixophyes* listed as endangered or vulnerable. Consequently, protocols for assisted reproduction, such as hormonal induction of ovulation have the potential to contribute to management and conservation of species across the genus. Currently, there is no data available for induced ovulation in gravid females of *Mixophyes* species. Adding to this, the group (Family: Myobatrachidae) is phylogenetically distant from many other amphibian taxa, and may consequently be quite different in its response to protocols that work well for other amphibian taxa, including its response to the injection of pituitary homogenates from unrelated species. Collectively the myobatrachids constitute about 50% of Australian amphibian species; 22% (n = 23) are considered vulnerable or endangered with 3 (2.8%) considered extinct; and they also constitute 48% of all threatened Australian frogs [47].

This paper reports the outcome of analyses of data on multiple ovulation and oviposition induction protocols employed on *Mixophyes fasciolatus* (the great barred frog). This species is the only non-threatened species of the *Mixophyes* in New South Wales and one of the few non-threatened species in Australia, and as such is an appropriate model for establishing protocols for assisted reproductive techniques (ART) for this genus. The data set does not contain all feasible protocols for the induction of ovulation (e.g. no data is available for effects of GnRHs or dopamine antagonists), but has focussed on the use of amphibian pituitary homogenates and mammalian FSH and LH preparations.

The results have implications for optimising oocyte collection in the other threatened *Mixophyes* species and, more broadly, other Australian ground (myobatrachid and limnodynastid) frogs. The results also demonstrate that, in species that respond to hCG, PMSG priming is effective in increasing the rate of ovulation (an assumption in some published protocols, not previously substantiated with data).

## Methods

Research described in this manuscript was undertaken following approval by University of Newcastle Animal Care and Ethics Committee which adheres to the NSW Animal Research Act, NSW Animal Research Regulation, and the Australian Code of Practice for the Care and Use of Animals for Scientific Purposes (706 06 08 to 706 06 10), and *M. fasciolatus* were collected under permit from the NSW National Parks and Wildlife Service.

### Sources and holding of animals

Adult female *Mixophyes fasciolatus* (great barred frogs) were collected from the mid to north coast regions of New South Wales, Australia (32^o^59’36.95”S; 151^o^26’44.23”E to 28^o^33’08.51”S; 153^o^18”31.72”E) during the spring to autumn periods (September to April) and held in groups of 10–12 in large plastic containers (approximately 1 m × 2 m × 1 m), with refuge sites provided as deep pine bark, leaf litter, wood and eucalypt bark. Access to water and food (brown crickets, *Acheta domestica)* was provided *ad libitum*, and environmental conditions (temperature and day length) were partially regulated by air-conditioning and fluorescent lighting in a facility that received partial lighting through external glass windows. Ambient temperatures varied between 16 and 28°C; light: dark cycle was approximately 10 L: 14 D.

Individual females were identified by implanted Passive Integrated Transponder (PIT) tags (GuangZhou HongTeng Barcode Technology Co. Ltd; Guangzhou). Females were assigned randomly to treatments (oviposition induction protocols), and females were used between the months of January and April over a period of two years. Induction protocols are described below. A proportion of females were subjected to induction attempts on more than one occasion, but were rested for at least 2 months between inductions (but generally longer than this, and up to 1 year). No prior information was available on either the seasonality of oogenesis, or the rate at which successive generations of mature oocytes are recruited to follicles in this species.

### Induction protocols

Gravid, adult females were subjected to a number of induction protocols, as indicated in Table 3. These were:

1. ***Controls*** given sham 250 μl saline injections (Simplified Amphibian Ringer (SAR); [6]) once per day for four days.

2. ***Varying cumulative doses of human chorionic gonadotropin (hCG)*** (Chorulon, Intervet (300 IU/ml); these were administered in the manufacturer’s diluent over up to three days (mostly over two days) in up to 4 aliquots to deliver the total doses indicated in Table 3.

3. ***hCG in combination with pituitary gland extracts*** from adult male cane toads (*Rhinella marina*); each female received pituitary extract injections on two consecutive days, each containing extracts from 3 pituitary glands in 250 μl of SAR, and total hCG injections (300 IU/ml in manufacturer’s diluent) over up to 3 days as indicated in Table 3.

4. ***hCG preceded with priming injections of pregnant mare serum gonadotropin*** Pregnant mare serum gonadotropin (PMSG) (Folligon, Intervet, 200 IU/ml in the manufacturer’s diluent) was administered between 2 and 6 days prior to the first hCG injection; 200 IU hCG was administered as two 100 IU aliquots administered separately on days 1 and 2 (Table 3), with the exception of a small number of females where the hCG injections were separated by 48 hours.

**Table 3:** **Oviposition by female*M. fasciolatus*after various mammalian gonadotropin treatments**

**Treatment groups**	**Day, time & number of females ovipositing**																					Oviposition (%)*
**Day**	**1**				**2**				**3**				**4**				**5**				**6**	
Light/dark (24 hr)**	**D**	**L**	**L**	**D**	**D**	**L**	**L**	**D**	**D**	**L**	**L**	**D**	**D**	**L**	**L**	**D**	**D**	**L**	**L**	**D**	**D**	
**(1) Control – Saline only: 4 injections**																						
**Saline**																						**0/6 (0%)**
**(2) hCG only**																						
**hCG 900**									**6**													**6/20 (30%)**
**hCG 1050-1400**									**1**				**1**	**1**	**1**							**4/17 (23.5%)**
**All hCG only**																						**10/37 (27%)**
**(3) hCG and pituitary glands**																						
**hCG 1200–1500 + 6 pituitaries**									**1**	**1**												**2/7 (29%)**
(4) PMSG and hCG (200 IU***)																						
**50 PMSG; -3 days**						**1**																**1/6 (17%)**
75 PMSG;-4, -2 days****										**4**												**4/12 (33%)**
75 PMSG;-5, -3 days****									**6**	**6**		**1**	**6**				**1**				**1**	**21/41 (51%)**
75 PMSG;-6, -4 days****					**1**			**1**	**1**				**1**	**1**			**1**					**6/9 (67%)**
**All PMSG and hCG (200 IU)**																						**32/68 (47%)**

Oviposition by female *M. fasciolatus* after either (1) sham injections with saline (2) injections with human chorionic gonadotropin (hCG) only (3) injections with human gonadotropin and 6 amphibian pituitary glands (*B. marinus*) (4) injection with pregnant mare serum gonadotropin (PMSG) indicated number of days before administration of first of two doses of human chorionic gonadotropin. * Number ovipositing/Number treated (% ovipositing) ** Light/Dark cycle = 12 hr L: 12 hr D; each L or D represents 6 hrs ***hCG = 100 IU administered on each of days 1 and 2. **** Where PMSG total dose = 75 IU: first dose = 50 IU; second dose = 25 IU administered on the indicated number of days before the first dose of hCG.

During the experimental period, females were maintained in individual vivaria on a 12 D: 12 L photoperiod at room temperature. Compounds were administered by injection into the dorsal lymph sacs via either a 23 or 26 G hypodermic needle.

### Multiple inductions

About 50% of the females were used in induction protocols on more than one occasion. Females used in more than one induction were rested for at least 2 months between induction attempts (but generally for longer), whether or not oviposition was observed in any preceding induction attempt. Females used in more than one induction attempt were randomly assigned to induction protocols on the second or third induction i.e. there was no systematic sequence, combination or set of induction protocols used where females were induced more than once. Thus, the data in Table 4 represents responses to multiple induction attempts employing various combinations of protocols (without systematic assignment).

**Table 4:** **Effect of diurnal cycle on timing of oviposition by*M. fasciolatus***

	**Treatment Group/Number of ovipositions observed**			
**Oviposition time (diurnal cycle)**	**hCG only**	**hCG and pituitary glands**	**PMSG and hCG**	**N**
0 – 12 hours*	9	2	30	41
12 – 24 hours**	1	0	2	3

Effect of diurnal cycle on timing of oviposition by *M. fasciolatus*. Data from Table 3. No significant effect of treatment group on timing of oviposition (p = 0.625); significant effect of stage of diurnal cycle on oviposition timing (p<0.0001). * 0–12 hours = 0:00–6:00 hr dark, 6:00–12:00 hr light; **12-24 hours = 12:00–18:00 hr light, 18:00–24:00 hr dark.

### Measures of response

Voluntary oviposition by females was used as the measure of response to induction protocols. Responses were recorded as occurring or not occurring. After injections, females were held in individual plastic vivaria for several days to assess their responses. Oviposition was recorded as oocytes deposited on the floor of vivaria; a small amount of isotonic saline being added to the vivaria around the time of oviposition to prevent desiccation of oocytes. Prior to oviposition, females were hydrated by small amounts of water added to the vivaria. Oocytes were not collected by manual stimulation. Most ovipositions involved the release of large numbers of oocytes (ranging from several hundred up to approximately 1000). A small number of ovipositions were associated with the release of fewer oocytes. The viability of several batches of oocytes was confirmed by *in vitro* fertilisation with sperm of male *M. fasciolatus*, with observations to the neurula stage. It is assumed in this study that oviposition indicates the occurrence of ovulation immediately prior to oviposition (as a result of the hormonal induction protocols), although no experiments were done to directly demonstrate this.

### Statistics

Data on the frequency of oviposition between treatments and treatment groups were analysed using non-parametric statistics. To avoid problems with expected frequencies less than 5, all frequency statistics were analysed using Fisher Exact Probability Tests ( http://vassarstats.net/; last accessed July 8, 2012), and employing the Freeman-Halton extension for contingency tables greater than 2 × 2. Data for the proportion of PMSG primed females ovipositing were plotted against time since first PMSG injection and the significance of the regression and correlation coefficients determined by least squares regression analysis.

## Results

### A comparison of protocols

***Controls (Group 1, Table***3***)*** No ovipositions were recorded in females subjected to saline only injections. This supports the conclusion that where ovipositions were recorded in hormonal treatment groups, these were a result of those treatments (p = 0.056, Table 5).

**Table 5:** **Matrix of Fisher exact test p values (one tailed) from data in Table**3

	**All treatments**	**hCG only**	**hCG and pituitary glands**	**PMSG and hCG**
**Saline Control**	0.056 (6,112)	0.182 (6,37)	0.269 (6, 7)	0.028 (6,68)
**hCG only**		-	0.628 (37,7)	0.035 (37,68)
**hCG and pituitary glands**		-	-	0.300 (7,68)

Matrix of Fisher exact test p values (one tailed) from data in Table 3 for comparisons between treatment groups of proportion of female *M. fasciolatus* ovipositing. Sample sizes of tested groups shown in parentheses.

***hCG alone (Group 2, Table***3***)*** Administration of hCG alone was shown to induce oviposition with doses totalling 900 to 1400 IU per female (Table 3). The overall oviposition rate in hCG only females was 27%. There was no evidence (p = 0.512) that increasing the total dose above 900 IU increased the rate of oviposition when hCG was the only treatment. hCG doses below 900 IU without the administration of other compounds were not tested in this study. Ovipositions with hCG alone were recorded up to 4 days after the initial injections, although most occurred by the end of the night of the second day.

***hCG and pituitary glands (Group 3, Table***3***)*** The overall oviposition rate in females treated with hCG and pituitary gland extracts was 29% (Table 3), which was not significantly different from the proportion of females ovipositing following hCG only treatments (p = 0.628, Table 5). No females were administered pituitary gland extracts without hCG in this study. All ovipositions occurred by the end of the night of the second day after the first injections.

***hCG following PMSG priming (Group 4, Table***3***)*** Some ovipositions with PMSG priming occurred earlier than any ovipositions with hCG alone. Taken together, the rate of oviposition in females subjected to PMSG priming prior to hCG injections (47%, Table 3) was significantly higher than in females subjected to hCG only (27%, Table 3; p = 0.035, Table 5). Ovipositions were recorded up to 6 days after the initial hCG injection, but most occurred from the morning of the third day through to the morning of the fourth day after the first hCG injection.

**Figure 1:**
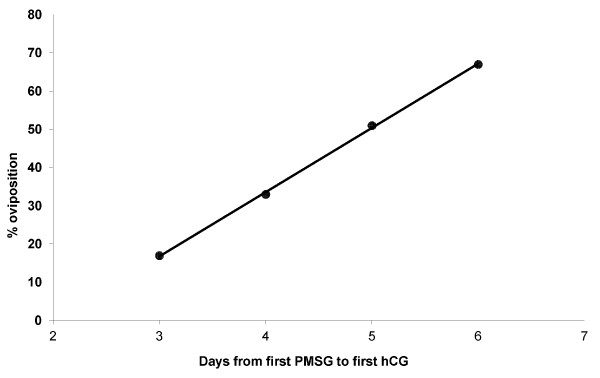
**Regression of mean% female*M. fasciolatus*ovipositing against time from first PMG injection to first hCG injection.** Data from Treatment Group “PMSG and hCG” in Table 3 (R^2^ = 0.9994; p<0.001).

There was a significant increase (p<0.001) in the rate of oviposition as a function of the time between the first PMSG injection and the first hCG injection, based on the regression of the percentage oviposition rate against the interval between first PMSG and first hCG (Figure 1) i.e. the oviposition rate was higher when PMSG priming began 5 to 6 days before first hCG than when it began closer to the first day of hCG administration.

### Repeated (multiple) inductions

Data from Table 6 shows the response of females to induction attempts performed on more than one occasion. 5/20 (25%) females in which inductions were attempted twice, ovulated twice. This indicates the capacity of females to go through multiple ovarian cycles and repeated ovulation and oviposition events in captivity. The total proportion of females subjected to induction protocols that oviposited at least once, increased with multiple induction attempts to 100% by the third attempt (Table 6); this trend of increasing proportion of females ovipositing at least once with repeated induction attempts indicates most, if not all, females held were in reproductive condition at some point, if not continuously over the duration of the study.

**Table 6:** **Proportions of female*M. fasciolatus*ovipositing after repeated induction attempts**

**Number of times induction attempted**	**1**	**2**	**3**
**Number of females**	29	20	6
**Number of females ovipositing at least once**	16	13	6
**% females ovipositing at least once**	55%^a^	65%^a,b^	100%^b^
**Number of females ovipositing twice**		5 (25%)	0

Capacity of female *M. fasciolatus* held under captive conditions to oviposit after induction attempts on more than one occasion. The data are pooled from all induction attempts across all protocols. Repeated attempts at induction of oviposition were separated by resting periods of at least 2 months. Percentages with different superscripts on the same row are significantly (p<0.05) different.

### Timing of oviposition

Oviposition occurred most frequently in the period including the second half of the dark period (0:00–6:00 hrs, Table 3) and the first half of the light period (6:00–12:00 hours, Table 3) of the daily photoperiod cycle, suggesting a behavioural orientation towards nocturnal oviposition (p<0.0001; Table 4) that may continue early into the light period.

## Discussion

This study adds *M. fasciolatus* to the small group of amphibian species (including *Xenopus laevis*, *Silurana tropicalis*, and some bufonids) that will ovulate and oviposit in response to protocols based only on mammalian gonadotropins. The range of hCG doses associated with successful oviposition in *M. fasciolatus* are similar to the range of doses reported in various *Xenopus/Silurana* protocols (this study up to 1400 IU, but optimised to 200 IU with PMSG priming; compared to *Xenopus/Silurana* 90 to 1000 IU without FSH/PMSG priming, 250 to 500 IU with priming; Table 1). The absolute dose for *M. fasciolatus* (not adjusted for body weight differences) is similar or less than that required to induce *B. americanus* and *B. fowleri* (Table 2). It is assumed that oviposition in this study also indicates ovulation occurring as a result of the induction process (as would be the case with most studies investigating the induction of oviposition in amphibians), although this was not tested directly; there are no published data on anurans known to the authors where ovulation and oviposition have been shown not to be linked, sequential events.

In *M. fasciolatus* there is a distinct benefit of priming with a gonadotropin with FSH activity. It was demonstrated in this study that the rate of ovulation can be increased from around 30% to more than 50% by priming females with PMSG. Interestingly, the benefits of priming were increased by extending the interval between the first PMSG priming dose and the first hCG dose. Based on the data from this study, the optimal protocol for inducing oviposition in *M. fasciolatus* involves injection of two priming doses of PMSG (50 IU and 25 IU) administered 6 and 4 days, respectively, prior to the injection of two ovulatory doses of 100 IU hCG, administered 24 hours apart.

Published induction protocols utilising hCG (Tables 1,2) identify *Xenopus* and *Silurana* species from the family Pipidae as those that respond most strongly to hCG, administered alone or in combination with a mammalian gonadotropin with FSH priming activity, or with other components of the hypothalamo-pituitary-gonadal pathway (Table 1). The data in Table 1 indicates a wide range of hCG concentrations (from 90 IU to 1000 IU in *X. laevis* as single hCG doses, a range of an order of magnitude) have been used to induce *Xenopus laevis* and *Silurana tropicalis*. An apparent higher sensitivity to hCG by *S. tropicalis* may reflect its smaller body size [48]. Various protocols in Table 1 have also reported benefits of priming the ovaries with either lower, anovulatory doses of hCG, or with PMSG to prime for maturation. Nevertheless, there do not appear to be reports of data on the generation of dose response curves for induction (hence the wide variation in protocols), even in *Xenopus*, which might indicate optimal doses that achieve maximum egg generation with the minimum hCG dose, nor which indicate the quantitative impacts of priming with hCG or PMSG. Most published *Xenopus/Silurana* protocols do not incorporate FSH priming (although a number of protocols have an hCG “priming” dose - see Table 1).

Although some *Xenopus/Silurana* protocols report priming doses of PMSG (Table 1), the authors of this study are unaware of a direct comparison in any study of protocols with and without priming that would prove a benefit of priming with FSH. This study thus provides the only published data that demonstrates in any amphibian species an increase in ovulation rate as a result of such priming, and that the maturational effect of priming increases with time since administration. FSH is common in mammalian protocols, recognising the role that FSH plays in recruiting follicles and priming the maturing follicle for ovulation by cumulus expansion during the LH induced resumption of meiosis and germinal vesicle breakdown [49,50]. LH but not FSH [51] causes a rise in progesterone in Xenopus follicles, indicating a separate, non-progesterone effect of FSH on follicle maturation.

Many anuran taxa, including various species of ranids, hylids and some bufonids (listed in Table 1) do not respond well (do not ovulate or ovulate at a very low rate) to induction with mammalian homologues of the amphibian gonadotropic hormones (a fact recognised many years ago: see [2,6]). The data in Table 2 shows that, in particular, ranids as a group are the least responsive to mammalian homologues of any higher amphibian taxon that has been studied in detail (resulting in the continued use of pituitary extracts for induction in this group, see below); bufonids are highly variable in their responses – many species have not been recorded as responding to mammalian homologues, others respond in conjunction with other effectors of the hypothalamic-pituitary-gonadal axis such as LHRH and progesterone, while at least two species (*B. americanus* and *B. fowleri*) respond reasonably well to hCG only inductions. Some less studied groups such as the Australasian hylids and the eleutherodactylids may or may not be poor responders, with results obtained on a small sample of species. In contrast, a number of urodeles appear to respond well to hCG.

Nevertheless, effective hCG doses for *Xenopus* and *Mixophyes fasciolatus* (allowing for differences in body weight) are high compared to doses reported to be effective in inducing ovulation in mammals (mouse 5 IU [52,53], dog 500 IU [54], rhesus monkey 4000 IU [55], cheetah 100–250 IU [56], cows 1500 to 2500 IU [57,58], mares 2,000 – 3,300 IU [59,60]; for example, the optimised dose of hCG used to induce oviposition in *M. fasciolatus* in this study (200 IU with PMSG priming) would have been sufficient to induce ovulation in 2 cheetahs [56]. This large differential in effective dose between mammals and responsive amphibians is also noted by Kouba and Vance [37] who observed that the ovulatory dose of hCG for *Xenopus* is 2000 times for that for the tiger. hCG is a convenient source of mammalian LH activity, but other mammalian sources are not necessarily more potent [8].

Given the challenges of inducing ovulation with mammalian homologues, pituitary extracts have continued to be used in many species to induce ovulation including bufonids [61], ranids [3,62,63] and at least one other myobatrachid [4]. No data were generated in this study on the capacity of amphibian pituitary gland extracts alone to induce ovulation and oviposition in *M. fasciolatus*, and there was no evidence (although the approach was not exhaustively investigated) of pituitary extracts potentiating the effects of the mammalian gonadotropins. The source of amphibian pituitary extracts in this study was *Rhinella marina*, which is not closely related to the *Mixophyes*[64]. The effect of homologous pituitary extracts remains untested in this species.

The results of this study do not preclude further work on this or other myobatrachid species to improve the rate and control of ovulation in assisted reproduction approaches. The use of gonadotropin releasing hormone analogues to induce ovulation has been reported in other myobatrachid species, *Pseudophryne guentheri*[65] and *P. corroboree*[66]. Investigations focussing on combining gonadotropin releasing hormones and dopamine antagonists [39] might also improve success rates and the control of timing of ovulation and oviposition in *M. fasciolatus* and other *Mixophyes* species, as might various combinations of hCG or other gonadotropins with progesterone [35,37].

*Mixophyes* frogs are one of the most threatened Australian myobatrachid genera (with only *Taudactylus* containing a higher proportion of threatened species). There is one instance of captive breeding reported in a *Mixophyes* species (*M. fasciolatus* at Melbourne Zoo; [67]). In the future, captive breeding approaches in this and other *Mixophyes* species may be augmented by assisted reproduction using techniques including induced ovulation for in vitro fertilisation that allow specific individuals to be paired for optimal genetic management, and improve efficiency and reduce costs in resource limited captive programs, and play a role in breeding programs selecting for disease resistance, such as against chytridiomycosis.

## Conclusions

This study found that *M. fasciolatus* is amongst the few amphibian species (including *Xenopus* (*Silurana*) and some bufonids) that respond well to the induction of ovulation utilising mammalian gonadotropins (hCG). The optimal protocol for *M. fasciolatus* involved two priming doses of PMSG (50 IU and 25 IU) administered at 6 and 4 days respectively, prior to two doses of hCG (100 IU), 24 hours apart. This study is also the first to demonstrate in an amphibian species that responds to mammalian gonadotropins that an increase in the ovulation rate occurs after priming with a gonadotropin (PMSG) with FSH activity.

## Abbreviations

hCG: Human chorionic gonadotropin; PMSG: Pregnant mare serum gonadotropin; LH: Luteinizing hormone; FSH: Follicle-stimulating hormone; GnRH: Gonadotropic releasing hormones; ART: Assisted reproductive techniques; PIT: Passive integrated transponder; SAR: Simplified amphibian ringer.

## Competing interests

The authors declare that they have no competing interests.

## Authors’ contributions

JC and SC contributed to the design of the study, participated in carrying out the trials, conducted the analysis of the data and interpretation of the findings, and prepared the manuscript. JG, AF and MM contributed to the design of the study, participated in carrying out the trials, and made comment on the manuscript. MA acquired funding for the study, contributed to the design of the study, made comment on the manuscript and helped with aspects of the laboratory work. All authors read and approved the final manuscript.
